# Insights Into the Major Processes Driving the Global Distribution of Copper in the Ocean From a Global Model

**DOI:** 10.1029/2019GB006280

**Published:** 2019-12-09

**Authors:** Camille Richon, Alessandro Tagliabue

**Affiliations:** ^1^ School of Environmental Sciences University of Liverpool Liverpool UK

**Keywords:** modeling, ocean, copper, trace metals, biogeochemistry, phytoplankton

## Abstract

Copper (Cu) is an unusual micronutrient as it can limit primary production but can also become toxic for growth and cellular functioning under high concentrations. Cu also displays an atypical linear profile, which will modulate its availability to marine microbes across the ocean. Multiple chemical forms of Cu coexist in seawater as dissolved species and understanding the main processes shaping the Cu biogeochemical cycling is hampered by key knowledge gaps. For instance, the drivers of its specific linear profile in seawater are unknown, and the bioavailable form of Cu for marine phytoplankton is debated. Here we developed a global 3‐D biogeochemical model of oceanic Cu within the NEMO/PISCES global model, which represents the global distribution of dissolved copper well. Using our model, we find that reversible scavenging of Cu by organic particles drives the dissolved Cu vertical profile and its distribution in the deep ocean. The low modeled inorganic copper (Cu') in the surface ocean means that Cu' cannot maintain phytoplankton cellular copper requirements within observed ranges. The global budget of oceanic Cu from our model suggests that its residence time may be shorter than previously estimated and provides a global perspective on Cu cycling and the main drivers of Cu biogeochemistry in different regions. Cu scavenging within particle microenvironments and uptake by denitrifying bacteria could be a significant component of Cu cycling in oxygen minimum zones.

## Key Points

- Phytoplankton uptake of ligand‐bound copper is necessary to meet phytoplankton copper requirements
- Reversible scavenging is responsible for the linear profile of dissolved copper
- Ligands are important drivers of the surface Cu cycling

## 1 Introduction

Copper (Cu) has a specific place among micronutrients; it is involved in many cellular reactions such as oxygen and iron acquisition (La Fontaine et al., 2002; Maldonado et al., 2006; Merchant & Helmann, 2012) but also denitrification (Granger & Ward, 2003), which results in bacteria and phytoplankton having an essential Cu requirement. However, high concentrations of copper in seawater can also induce toxic effects (Brand et al., 1986; Debelius et al., 2011; Moffett et al., 1997), with a deleterious impact on growth. For this reason, copper is often referred to as a “Goldilocks” element, whereby there is a balance between enough Cu to avoid growth limitation and not too much as to induce toxicity. Understanding the role of copper as an essential micronutrient or toxin requires knowledge of the major processes shaping the availability of copper in space and time, most notably encompassed in the vertical profile. Uptake of Cu will be affected by its bioavailability, which is also affected by the chemical speciation of Cu. Cu is also of interest because of its emerging linkages to iron cycling and acquisition (e.g., Maldonado et al., 2006; Peers & Price, 2006; Wood, 1978). Newly available data sets concerning the large‐scale distribution of Cu in the ocean are emerging as part of the GEOTRACES program (e.g., Schlitzer et al., 2018) and reveal the key features of its oceanic distribution. For instance, Cu concentrations are higher in the surface coastal Pacific and Atlantic oceans, potentially due to a combination of river discharge and aerosol deposition (Boiteau et al., 2016; Jacquot & Moffett, 2015; Roshan & Wu, 2015). In the Southern Ocean, surface Cu concentrations are high in winter, and the major processes driving their variability in the surface layer are suggested to be phytoplankton uptake and mixed layer depth variations (Cloete et al., 2018; Ellwood, 2008). A key unknown remains the unusual linear profile of Cu with respect to depth, which typifies profiles across the Atlantic and Pacific Oceans (Heller & Croot, 2015; Jacquot & Moffett, 2015; Roshan & Wu, 2015). Specifically, the relative role played by regeneration of organic material, interior sources, and scavenging and the role of organic complexation of Cu with ligands in shaping the linear Cu profile remain poorly constrained globally.

The vertical distribution of Cu shows a notable difference to “nutrient‐like” or “scavenged” elements, being typified by a linear increase with depth (Tagliabue, 2019). To explain the widespread linear increase of dissolved Cu concentrations with depth, Hines et al. (1984) and Biller and Bruland (2013) hypothesized the presence of deep water sources of dissolved Cu from sediments or hydrothermal vents. However, it has been suggested that Cu from these sources would be associated with high particle loads which would likely scavenge Cu back to the sea floor, having very little impact on the wider water column (Jacquot & Moffett, 2015; German et al., 1991; Roshan & Wu, 2015). Moreover, if hydrothermal and sedimentary sources were the only driver of Cu vertical profiles, the linearity might be expected to only occur in specific areas of the ocean due to the noted regionality in hydrothermal signals (Tagliabue & Resing, 2016). Alternatively, Little et al. (2013) hypothesized the existence of reversible scavenging of Cu onto particles as an explanation for the Cu vertical profile. As also suggested for zinc (Weber et al., 2018), high particle concentrations in surface seawater lead to high scavenging of dissolved phases onto particles, and the decreasing particle concentrations with depth then promote the release from particle phases. Although a reversible scavenging model brings a theoretical solution to the linear Cu profile, it has not been tested globally.

The chemical speciation of Cu in seawater is a key component of its oceanic cycling with more than 99% of dissolved Cu (DCu) in the surface being organically bound with ligands (Coale & Bruland, 2003; Jacquot & Moffett, 2015), and inorganic copper (Cu') is only a very small fraction. Many different compounds form the ligand pool in seawater, and Cu ligands are usually grouped into two categories: strong ligands (with conditional stability constants, KL for the reaction Cu' + L = CuL, over 10^13.5^) usually produced by bacteria and plankton (Moffett & Brand, 1996) and weak ligands (KL lower than 10^13.5^) that are usually derived from organic matter originated from sea or from land (Whitby et al., 2018). Strong ligands are often thiol groups (among which glutathione is the most represented) and are produced by microorganisms, in particular diatoms, in response to elevated Cu' concentrations to detoxify copper (Leal et al., 1999; Whitby et al., 2018). Because of the complexity of the chemical identification of ligands, their role in oceanic Cu biogeochemical cycling is difficult to characterize.

The organic speciation of Cu may control its bioavailability to phytoplankton. Because of its important physiological role (e.g., for iron, oxygen, and nitrogen cycling; Granger & Ward, 2003; La Fontaine et al., 2002; Maldonado et al., 2006; Merchant & Helmann, 2012), Cu is required in phytoplankton cells in proportions varying on average between 0.4 and 2 mmol:mol relative to P and up to 3 mmolCu:molP (Twining & Baines, 2013; Twining et al., 2019). The most bioavailable form of copper for phytoplankton is Cu', but the very low concentrations in surface seawater may indicate that insufficient Cu' is available to meet phytoplankton requirements. Semeniuk et al. (2009) suggest that ligand‐bound copper can be at least partly available to phytoplankton and bacteria. Whether dissolved Cu distributions and cellular quotas can be reconciled with Cu' as the only bioavailable form remains untested.

In this study, we present a global 3‐D coupled physical‐biogechemical ocean model of copper, implemented in the state‐of‐the‐art NEMO/PISCES platform that simulates global biogeochemical cycling (Aumont et al., 2015; Tagliabue et al., 2018) to examine the role of different external sources and internal cycling in regulating the distribution of Cu and its bioavailability. We describe the model main equations, physical and biogeochemical forcings, initial conditions, and the different experiments in section 2. Section 3 presents an evaluation of the model results with available measurements. This section also provides evidence that reversible scavenging is the main driver of the dissolved Cu linear profile in the global ocean and that ligand‐bound Cu is at least partially bioavailable for phytoplankton. Section 3.4 presents a global budget of oceanic Cu including all biogeochemical reactions and fluxes. Section 4 puts forward a holistic view of Cu cycling based on our model, which highlights the major processes and sources impacting Cu in all ocean regions. Finally, section 5 provides an analysis of Cu cycling in the Pacific oxygen minimum zone (OMZ) and tries to identify the key processes responsible for the [DCu] observed in the region.

## 2 Methods

### 2.1 Model Description

We embedded a 3‐D model of Cu biogeochemistry within the widely used PISCES model (Aumont et al., 2015), coupled to the dynamical model NEMO (Madec, 2006). PISCES reproduces the biogeochemical cycling of various macronutrients (phosphate PO_4_, nitrate NO_3_, ammonium NH_4_, and silicate Si), planktons (nanophytoplankton, diatoms, microzooplankton, and mesozooplankton), and one trace element (iron [Fe]). Recent developments allowed representing new trace elements such as manganese (Mn; Hulten et al., 2017) and cobalt (Co; Tagliabue et al., 2018) and gave new insights on the processes governing Fe distributions in the ocean (Tagliabue & Resing, 2016). Seven new tracers were implemented in PISCES in order to represent copper biogeochemical cycling: dissolved copper (DCu); scavenged copper, which represents copper adsorbed on the surface of particles (SCu, divided into small and large scavenged particles, SCup and SCug), this copper can be desorbed to the dissolved phase at depth; copper associated with biogenic particles (CuPart, divided into small and large copper particles, pCuPart and gCuPart) that can only be resupplied via bacterial activity; and copper in phytoplankton cells (Cu^*ϕ*^, with *ϕ* = N for nanophytoplankton or *ϕ* = D for diatoms). All parameter values are summarized in Table 1. Other biogeochemical parameters of PISCES are found in Aumont et al. (2015).

**Table 1:** Summary of the Cu Model Parameters

Name	Description (units)	Value	Reference
Sol_Cu_	Solubility of aerosol Cu	0.40	Paytan et al. (2009)
curat	Cu:C in zooplankton (μmolCu:molC)	10	This study
ksCu_*ϕ*_	Half saturation constant for DCu uptake (nmolCu/L)	4 and 12	This study and Guo et al. (2010)
*θ*_max_	Maximum Cu:P in phytoplankton (molCu:molP)	2E‐3	Twining and Baines (2013)
KDCug	Partition coefficient for Cu scavenging on big particles (1/mmol)	5E‐3	This study
KDCup	Partition coefficient for Cu scavenging on small particles (1/mmol)	100E‐3	This study
KL	Cu ligand binding strength	10^13.5^	Whitby et al. (2018)

*Note*. When two values are given, the first is for nanophytoplankton, and the second is for diatoms.

#### 2.1.1 General Equations

The general equation for DCu cycling is presented in equation (1):

(1) $$\frac{\delta DCu}{\delta t}=Riv_{\text{Cu}}+Aero_{\text{Cu}}-Up_{\text{Cu}}-Scav_{\text{Cu}}+Remin_{\text{Cu}}+Recycling_{\text{Cu}}.$$

DCu is the sum of the dissolved forms of copper in seawater: Cu' and ligand‐bound copper (CuL). Ligands can represent a variety of compounds with a wide range of Cu affinity. As a first approach, we use a simple ligand model with one type of ligand (L), uniformly distributed over the ocean ([L] = 1 nM) and a fixed complexation constant (KL) of 10^13.5^, representing a bulk average of all ligand types for the entire water column.

*Riv*_Cu_ and *Aero*_Cu_ represent Cu from external sources (rivers and aerosols, respectively). *Up*_Cu_ is Cu uptake by phytoplankton, *Recycling*_Cu_ is recycling of dissolved Cu by zooplankton, *Scav*_Cu_ represents the scavenging flux, and *Remin*_Cu_ is bacterial remineralization from particulate material.

The general equation for scavenged Cu is presented in equation (2):

(2) $$\frac{\delta SCu}{\delta t}=Scav_{\text{Cu}}-sinking,$$

with *Scav*_Cu_ as copper scavenging and *sinking* as SCu sinking rate, increasing with depth following the same equations as C, Fe, and Co (Tagliabue et al., 2018).

The general equation for Cu^*ϕ*^ is presented in equation (3):

(3) $$\frac{\delta Cu^{\phi }}{\delta t}=Up_{\text{Cu}}-m^{\phi }-Graz_{\rho }^{\phi },$$

with *m* ^*ϕ*^ as phytoplankton mortality and $Graz_{\rho }^{\phi }$ as grazing of zooplankton species *ρ* on phytoplankton species *ϕ* (with *ρ* = Z for microzooplankton and *ρ* = M for mesozooplankton). Calculation of these terms is described in Aumont et al. (2015).

The general equation for CuPart is presented in equation (4):

(4) $$\frac{\delta CuPart}{\delta t}=Scav_{\text{Cu}}+Excr_{\text{Cu}}+Agg_{\text{Cu}}-Remin_{\text{Cu}},$$

with *Agg*_Cu_ as the aggregation term, which is a positive function of particulate organic matter (see Aumont et al., 2015), and *Excr*_Cu_ the excretion of Cu via zooplankton fecal pellets.

The biogeochemical parameter values are based on the PISCES general equations valid for macronutrients (Aumont et al., 2015), Fe (Tagliabue & Resing, 2016), and Co (Tagliabue et al., 2018).

#### 2.1.2 External Sources of Copper

We derive riverine inputs of DCu from the Fe:Cu ratio in rivers based on Gaillardet et al. (2014). Cu flux is computed in PISCES similarly to the other nutrient fluxes (see Aumont et al., 2015).

Atmospheric deposition of natural and anthropogenic Cu is derived from the modeled deposition fluxes of Paytan et al. (2009). Solubility of Cu from atmospheric deposition is fixed to 40% as an average for all aerosol types represented in the atmospheric model (see also Sholkovitz et al., 2010, for a discussion on aerosol Cu solubility), and dissolution is considered instantaneous upon deposition on the surface ocean. Atmospheric Cu deposition is added to the DCu pool according to the following equation:

(5) $$[DCu]=[DCu]+\mu_{\text{Cu}}\times Sol_{\text{Cu}},$$

with *μ*_Cu_ as the total (dry + wet) atmospheric Cu flux and Sol_Cu_ the solubility. Atmospheric Cu fluxes show a satisfying correlation with measurements (see Mahowald et al., 2018, Figures 4e and 4f). No Cu input from hydrothermal vents or from sediments are considered in this model.

#### 2.1.3 Reversible Scavenging Model

We simulate reversible Cu scavenging onto organic particles, similar to zinc (Weber et al., 2018). This model assumes a continuous exchange between the inorganic copper (Cu') and organic particles, instead of the irreversible uptake of trace metals onto particles, which is used to represent most trace metals (see Bacon & Anderson, 1982). We assume a fast equilibrium between adsorption and desorption, allowing us to describe Cu scavenging as a function of the organic particles concentration (*Part*_*C*_) and a partition coefficient (*KDCu*). The equation for scavenging is presented in equation (6).

(6) $$Scav_{\text{Cu}}=\frac{ztrc}{(ztrc+1)\times Cu^{\prime }}-SCu,$$

(7) $$ztrc=Part_{C}*KDCu.$$

The strong KL for copper‐ligand complexation keeps [Cu'] low in the water column. Therefore, the term on the right‐hand side of equation (6) is positively correlated with *Part*_*C*_. When this term is greater than 0, net scavenging is occurring. On the contrary, if the particle load (*Part*_*C*_) is low, resolubilization is occurring (Cu' release from the scavenged pool).

#### 2.1.4 Copper Biological Uptake

Cu uptake by phytoplankton is modeled following the Co model from Tagliabue et al. (2018) and is represented using an evolving Cu:P ratio in the planktonic cells (equation (8)).

(8) $$Up_{\text{Cu}}=\mu_{\max}^{\phi }\times \theta_{\max}^{\phi }\times \frac{bCu}{bCu+ksCu_{\phi }}\times \frac{1-\theta^{\phi }/\theta_{\max}^{\phi }}{1.05-\theta^{\phi }/\theta_{\max}^{\phi }}.$$

In this equation, $\mu_{\max}^{\phi }$ is the maximum phytoplankton growth rate and is fixed to 1.05 day^−1^ for both nanophytoplankton and diatoms (Aumont et al., 2015). *θ* ^*ϕ*^ is the Cu:P ratio and $\theta_{\max}^{\phi }$ the maximum ratio (see Table 1 and Twining & Baines, 2013). *bCu* is the bioavailable Cu concentration (bioavailable Cu can be all DCu or Cu' alone) and *ksCu*_*ϕ*_ the half saturation constant (expressed for DCu in nmolCu/L for Cu uptake) for phytoplankton group *ϕ*. Following equation (8), Cu uptake is down‐regulated when Cu:P gets close to the maximum value.

### 2.2 Experimental Design

After a 500‐year spin‐up, all simulations ran for 600 years starting from the same initial conditions. The reference simulation is called REF and two sets of experiments were performed (SCAV and INORGANIC‐CU). SCAV is designed to quantify the effects of different partition coefficients (KDCu) on copper vertical distribution (LOWSCAV and HIGHSCAV). FESCAV serves as a control to show that reversible scavenging is responsible for the linear [DCu] profile over the ocean. In this simulation, Cu scavenging is similar to iron scavenging: once Cu' is adsorbed onto particles, it can only be remobilized by bacterial activity or recycled after grazing by zooplankton. The aim of the INORGANIC‐CU and INORGANIC‐CU2 simulations is to observe whether phytoplankton can maintain their Cu:P quotas if they were only using Cu' as a Cu source instead of using Cu' and CuL as in REF. INORGANIC‐CU2 aims at maximizing the uptake of Cu' by phytoplankton by lowering phytoplankton half saturation constants for Cu' uptake. All simulations and their specific parametrizations are described in Table 2.

**Table 2:** Description of all simulations

Name	KDCup	KDCug	Rev. Scav.	ksCu_*N*_	ksCu_*D*_	bCu
REF	**100E‐3**	**5E‐3**	**Yes**	**4**	**12**	**DCu**
LOWSCAV	**50E‐3**	**1E‐3**	**Yes**	4	12	DCu
HIGHSCAV	**200E‐3**	**10E‐3**	**Yes**	4	12	DCu
FESCAV	**100E‐3**	**5E‐3**	**No**	4	12	DCu
INORGANIC‐CU	100E‐3	5E‐3	Yes	**4**	**12**	**Cu'**
INORGANIC‐CU2	100E‐3	5E‐3	Yes	**0.01**	**0.03**	**Cu'**

## 3 Results and Discussion

### 3.1 Dissolved Copper Distribution

**Figure 1:**
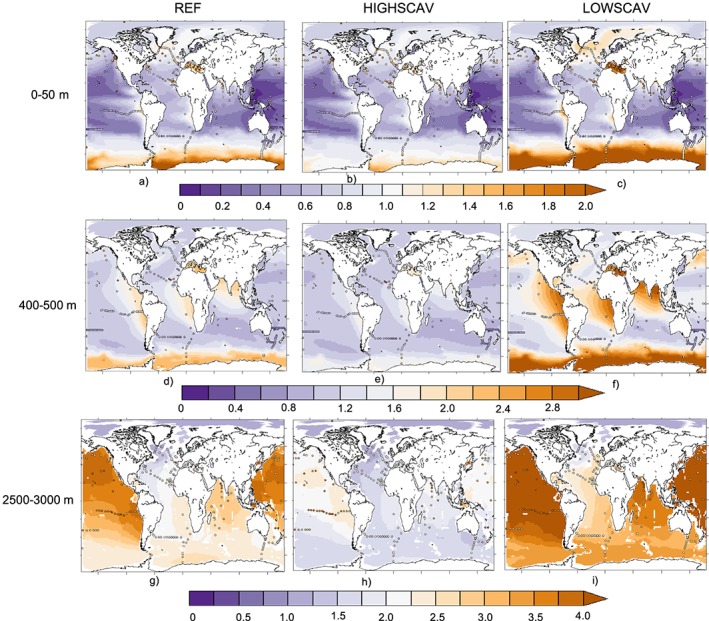
Average DCu concentration (nmol/L) from the REF (a, d, g), HIGHSCAV (b, e, h), and LOWSCAV (c, f, i) simulations in the 0–50 m (a–c), 400–500 m (d–f), and 2,500–3,000 m (g–i) depth layers. Dots represent data points.

**Figure B1:**
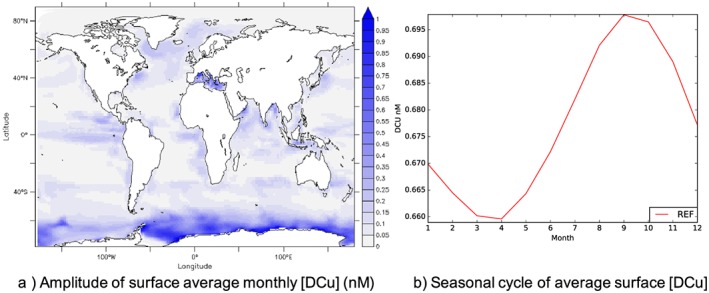
(a) Amplitude of average monthly [DCu] in surface (0–50m). (b) Seasonal cycle of average surface (0–50 m) [DCu] over the global ocean.

The modeled DCu distribution in our reference simulation (REF) is able to closely reproduce measurements from different campaigns and experiments (A. Gourain personal communication compiled data from, e.g., the GEOTRACES database, Schlitzer et al., 2018; line P transect in the North Pacific, Posacka et al., 2017; Semeniuk et al., 2016; and PINTS expedition in the Tasman Sea, Hassler et al., 2014; see Figures 1a, 1d, and 1g). Average [DCu] in the first 50 m is 0.83 nmolCu/L, with minimal concentrations (below 0.20 nmolCu/L) in the subtropical Pacific along the Indonesian coasts and in the subtropical oligotrophic gyres and maximal concentrations (over 1.5 nmolCu/L) in the Southern Ocean where modeled [DCu] slightly overestimates the measurements. However, most of the measurements in this region are obtained during summer, which may explain the lower annual mean in our model. An assessment of the seasonal variability in our model showed that surface [DCu] can vary by up to 1 nmolCu/L in this region (see Figure B1). In the intermediate layer (400–500m), the model overestimates [DCu] by around 40% in the Pacific OMZ. In the deep ocean, concentrations in the North Pacific are measured between 2 and 3 nmolCu/L, and the model values are over 3 to 4 nmolCu/L.

**Figure 2:**
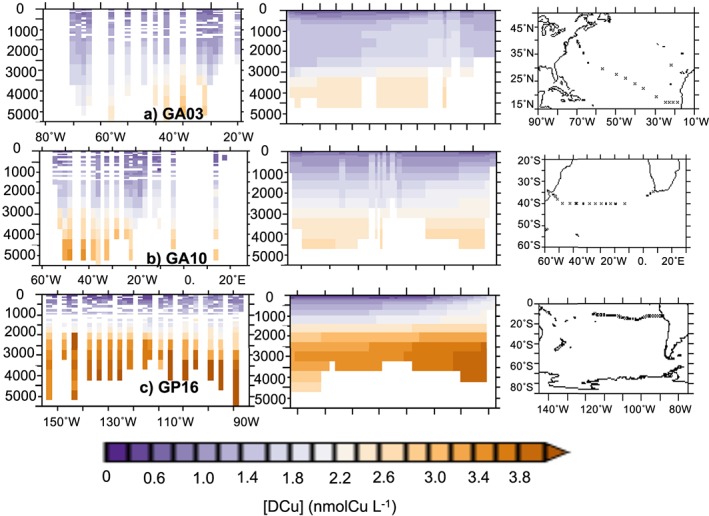
Average [DCu] (nmol/L) along the GA03 (a), GP10 (b), and GP16 (c) GEOTRACES cruises. Left panels represent the data, middle panels the REF simulation, and right panels cruise tracks.

Overall, modeled [DCu] is coherent with the measurements of the vertical DCu distribution along the GA03 (Roshan & Wu, 2015), GA10 (Little et al., 2018), and GP16 (Boiteau et al., 2016) GEOTRACES sections as represented in Figure 2. The REF simulation reproduces the general feature of a linear increase of [DCu] with depth for all sections. [DCu] in the Pacific OMZ is overestimated (Section GP16, between 400‐ and 700‐m depth; Figure 1d). Along GP16, the [DCu] increase with depth is too strong, leading to overestimated concentrations between 500 and 3,000 m. In the deep waters of the North Atlantic (Section GA03; Figure 2a), [DCu] is well represented in the western sector (between 20° and 40°W) but overestimated by around 0.6 to 0.8 nmolCu/L in the eastern sector.

**Figure 3:**
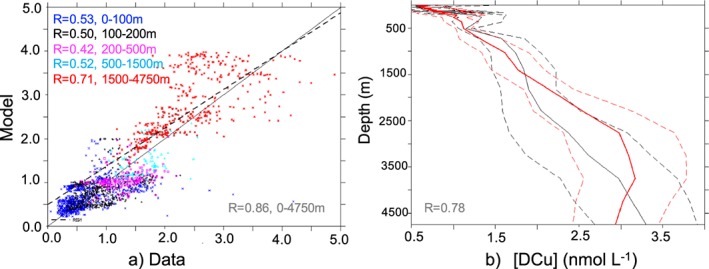
(a) Scatter plot of DCu concentration (nmol/L) in the model versus measured concentrations; the black line represents the 1:1 line, and the dotted line represents the slope of the model versus data regression. Numbers in the left corner represent the correlation coefficient (*R*, log‐log regression) and the depth range; numbers in the left corner represent values for the entire water column. Statistics are calculated on the log of values. Colored dots represent different depth ranges. The number of observations per depth range is 0–100 m, 924; 100–200 m, 532; 200–500 m, 222; 500–1,500 m, 180; and 1,500–4,750 m, 497. (b) Average [DCu] profiles from the data (black line) and from the model (red line). Dashed lines represent the standard deviation, and number on the left corner represents correlation coefficient between the model and data.

When evaluated statistically, the REF model reproduces the observations well across different depth ranges (Figure 3a) with a global correlation of 0.86, and the model‐data regression line (slope = 0.92) is very close to the 1:1 line. The weaker model performances between 200 and 500 m (*R*=0.42) highlight the model deficiencies in the eastern Pacific OMZ in particular. Concerning the vertical profile, when the model is compared to the data at the same vertical coordinates, it remains within the observation variability and reproduces the progressive DCu increase with depth well (Figure 3b). Overall, the REF simulation is a solid foundation from which to assess the main processes driving the bioavailability and distribution of Cu in the ocean.

### 3.2 The Role of Reversible Scavenging

**Figure 4:**
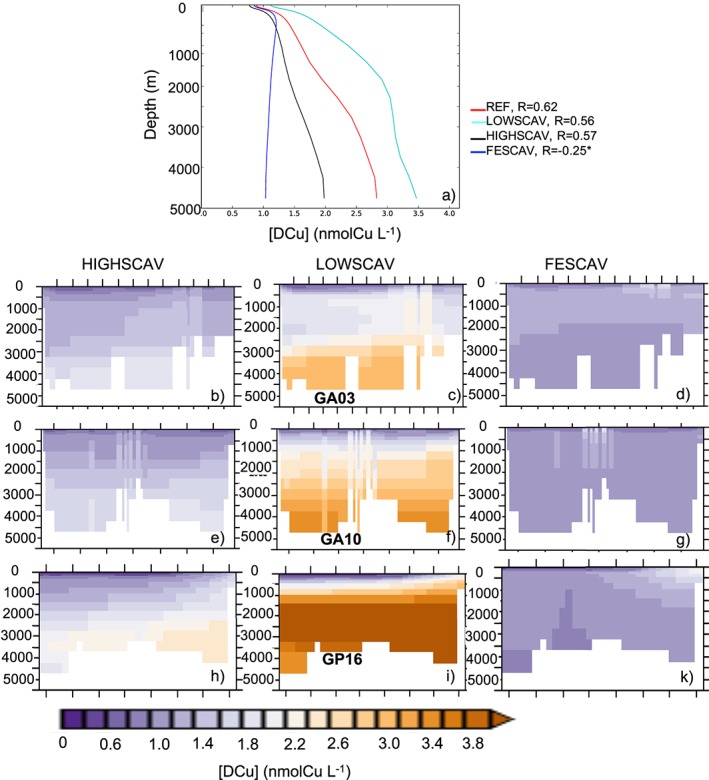
(a) Average profiles of [DCu] in the global ocean from REF and all the SCAV simulations. Numbers represent the regression fit (*R*) between [DCu] and depth; the star indicates that the statistical regression is not significant (*p* value > 1%). The (b) to (k) represent [DCu] in HIGHSCAV (b, e, and h), LOWSCAV (c, f, and i), and FESCAV (d, g, and k) along the GA03, GA10, and GP16 cruise sections. See Figure 2 for the data and REF concentrations.

The simulations LOWSCAV, HIGHSCAV, and FESCAV examine the effects of different scavenging modes and partition coefficients on [DCu] vertical distribution. Figure 4a shows how reversible scavenging (simulations REF, HIGHSCAV, and LOWSCAV) is responsible for the linear profile of [DCu]. The average vertical DCu profile in REF can be modeled by a linear regression with *R*=0.62 and *p* value < 1%. Reducing or enhancing the scavenging partition coefficient as in HIGHSCAV or LOWSCAV modifies the slope of the regression accordingly and systematically degrades the regression coefficient (*R*) in comparison to REF. The high partition coefficients in HIGHSCAV results in Cu' being quickly adsorbed onto particles and removed from the water column via sinking, leading to underestimated [DCu] over the water column by about 1 nmolCu/L (see Figures 1b, 1e, 1h, 4b, 4e, and 4h). On the other hand, the low partition coefficients in LOWSCAV leads to overestimated [DCu] (see Figures 1c, 1f, 1i, 4c, 4f, and 4i) and increases the average deep [DCu] by about 0.6 nmolCu/L. The iron‐like scavenging represented in FESCAV is the most common form of trace metal scavenging and leads to a nutrient‐like [DCu] profile across the global ocean, with uniform concentrations around 1 nmolCu/L below the euphotic layer.

Overall, [DCu] in the surface ocean is not highly impacted by the scavenging partition coefficient (see Figures 1a–1c); however, the partition coefficient impacts [DCu] in the OMZ regions, and the anomalies propagate to the deep ocean layers, affecting the entire water column (see Figure 1d to 1i).

### 3.3 Bioavailable Form of Copper

**Figure 5:**
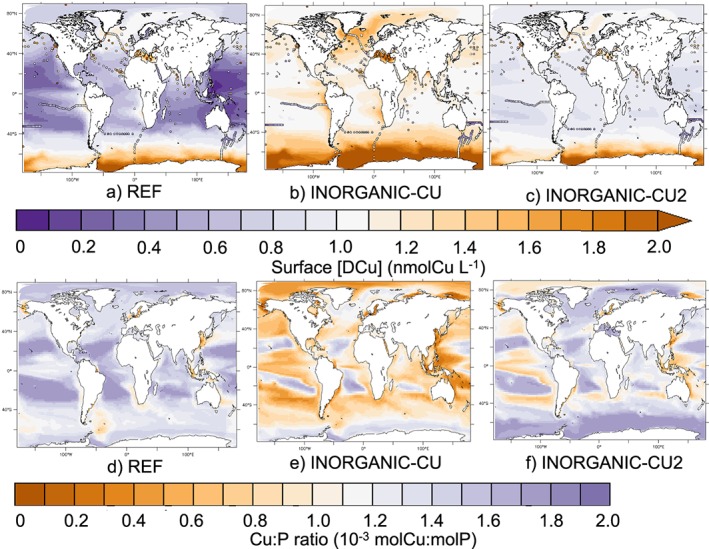
(a–c) Maps of [DCu] in surface (0–100 m) in REF and in the INORGANIC‐CU and INORGANIC‐CU2 simulations (background) and in the GEOTRACES data (circles). (d–f) Maps of the average Cu:P ratio (mmolCu:molP) in phytoplankton cells (0–100 m).

We conducted a set of simulations to assess how different assumptions regarding phytoplankton uptake impacts DCu distribution in the ocean. The total Cu' pool in the global ocean is 83 times smaller than total DCu in the REF simulation. In agreement, Coale and Bruland (2003) and Moffett et al. (1997) observed that over 99% of DCu is bound with organic ligands. Figure 5d shows that, in REF, equatorial and high latitudes Cu:P quotas are around 1.2 to 1.5 mmolCu:molP. Around the East Asian coasts, the Baltic Sea, the Bering Strait, and the Drake Passage, the cellular Cu:P ratio is below 1 mmolCu:molP. Phytoplankton Cu:P in REF is close to the maximal value of 2 mmolCu:molP in most oceanic regions, indicating that phytoplankton is able to satisfy its copper demand. We also calculated the total Cu uptake in the first 100 m of the global ocean and found a total uptake of 31 GmolCu/year. Converted into picomoles of Cu per day per liter, results from REF give 2.4 pmolCu·day^−1^·L^−1^, which is on the lower end of Semeniuk et al. (2009) and Semeniuk et al. (2016). These authors also found variability in uptake rates in the northwestern Pacific (between 3 and 125 pmolCu·day^−1^·L^−1^).

In INORGANIC‐CU, reducing the bioavailable Cu pool to only Cu' leads to only 50% to 80% ligand‐bound copper and high surface [DCu] in all oceanic regions, including the oligotrophic gyres where it is close to the ligands concentration (around 1 nmol/L; Figure 5). Improving the ability of phytoplankton to acquire Cu' at low concentrations by reducing the ksCu_*ϕ*_ to 0.01 and 0.03 nmol /L in the INORGANIC‐CU2 simulation brings the proportion of ligand‐bound copper over 95% in most parts of the ocean. However, there is, to our knowledge, no published value of phytoplankton half saturation constant for Cu. However, Figure 5c shows that reducing ksCu_*ϕ*_ still leads to up to 50% overestimation in surface [DCu] and low spatial variability as high levels of CuL remains unused in the surface ocean. Figures 5e and 5f show that relying on Cu' as the only Cu source decreases the Cu:P ratio in the phytoplankton cells below that of REF, even with a very high phytoplankton affinity for Cu (INORGANIC‐CU2). In the equatorial and high latitudes regions, Cu:P quotas are around 0.5 to 1.5 mmolCu:molP in INORGANIC‐CU2 and below 0.5–1 mmolCu:molP in INORGANIC‐CU. Around the East Asian coasts, the Baltic Sea, the Bering Strait, and the Drake Passage, the cellular Cu:P ratio is below 0.5 mmolCu:molP in INORGANIC‐CU2 and is close to 0 in INORGANIC‐CU. These results indicate that the surface ocean Cu' pool is too small to fuel phytoplankton cells to their maximal Cu:P quota, even with very low ksCu_*ϕ*_. Therefore, at least a fraction of CuL has to be bioavailable to phytoplankton in order to avoid submaximal quotas in phytoplankton cells that may have consequences on cellular functions (see Annett et al., 2008). Finally, Cu uptake rate decreases in INORGANIC‐CU2 to 27 GmolCu/year (2.0 pmolCu·day^−1^·L^−1^), which is below Semeniuk's estimates, indicating that Cu' is a too small pool to maintain Cu biogeochemical cycling in the surface ocean.

Accurately representing Cu bioavailability and uptake in our model affects the Cu distribution and phytoplankton cellular ratios (Figure 5). Neither limiting nor toxic effects of Cu on phytoplankton and zooplankton growth are included in the present model configurations. Laboratory assessments of Lowest Observed Effect Concentration and No Observed Effect Concentration, which are necessary to assess toxic Cu concentrations, are rare (see, e.g., Suratno et al., 2015). Representing both the limitation and toxicity effects of Cu on plankton growth in PISCES relies on deeper knowledge on physiological effects of Cu and on estimations of concentration thresholds for limiting and toxic effects (Prosnier et al., 2015). However, such developments should be the next step toward modeling the potential impacts of Cu and other metal contamination in the ocean food webs.

### 3.4 Global Budget and Residence Time of Oceanic Copper

**Figure 6:**
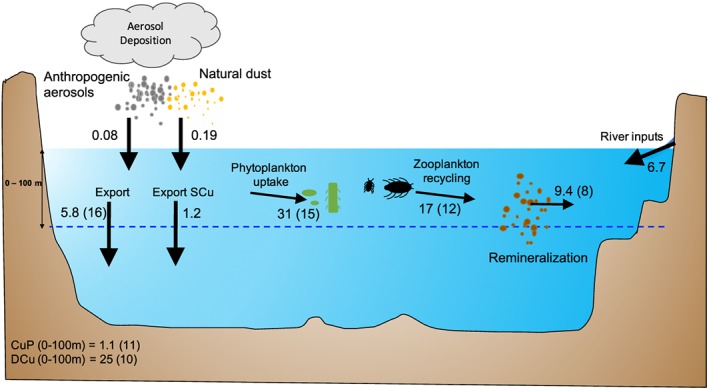
Cu budgets in the first 100 m of the global ocean. Numbers represent Cu fluxes (in GmolCu/year); numbers in the left corner represent total inorganic particulate and dissolved Cu (CuPart and DCu) in the first 100 m (GmolCu). Numbers in parentheses represent the Cu:C ratio of each process and compartment (μmolCu:molC)

Cu supply to the ocean is dominated by rivers, with aerosols playing a minor role, leading to a residence time of 400–500 years (Figure 6). Natural aerosols are the dominant aerosol Cu source at the global scale (respectively, 0.08 and 0.19 GmolCu/year of anthropogenic and natural Cu aerosols; see Figure 6). However, our estimates of aerosol Cu deposition based on Paytan et al.'s (2009) modeling study are 50% higher than Little et al.'s (2014) estimate of 0.054 GmolCu/year based on a global average Cu deposition flux and average solubility.

The global Cu riverine flux from our model is estimated at about 6.7 GmolCu/year, which is about 10 times higher than Little et al.'s (2014) estimate of 0.6–0.8 GmolCu/year, based river isotopic composition from Vance et al. (2008) with few sampling points. Our Cu river flux estimation is based on a fixed Cu:Fe ratio in rivers from Gaillardet et al. (2014) who considered a greater number of samples but used only measurements in rivers, far from anthropogenic activities, and may therefore underestimate the total Cu river flux. Moreover, river catchment basins are often rich in organic matter, humics, and ligands that may bind copper and modify its bioavailability, but there is no global estimate of ligand fluxes from rivers. In spite of these potential caveats, our estimation agrees with Little et al. (2014) that rivers are the main external Cu source to the global ocean. However, our greater input fluxes of Cu result in a much shorter residence time for Cu of between 400 and 500 years.

Upon arrival in the surface ocean, a fraction of Cu is scavenged by particles and sinks into deeper water, representing a loss of 1.2 GmolCu/year from the top 100 m of the global ocean. In contrast, phytoplankton uptake represents a sink of DCu of 31 GmolCu/year, with approximately half of the uptake flux being recycled by zooplankton (17 GmolCu/year), and 9 GmolCu/year is remineralized by bacteria. The model suggests a residence time of 3 years for the top 100 m, which agrees with estimates of 2.5–8 years from the North Pacific (Semeniuk et al., 2016). The remainder 5.8 GmolCu/year sinks into deeper water as particulate organic Cu.

**Figure A1:**
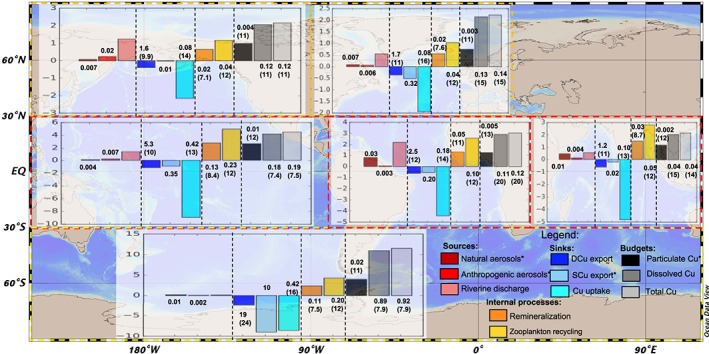
Barplot of total Cu budgets and fluxes in the first 100 m (in Gmol/year) in different oceanic regions. High latitudes: >60°N or <60°S. Midlatitudes: between 30° and 60° N or S. Equatorial: between 30°S and 30°N. The numbers above the bars represent the average fluxes or concentrations. Numbers in parentheses are the Cu:C ratio of the component (10^−6^ molCu:molC). Aerosol deposition and export fluxes are given in 10^−6^ molCu·m^−2^·year^−1^; phytoplankton uptake, remineralization, and zooplankton recycling are given in 10^−6^ molCu·m^−3^·year^−1^; particulate, dissolved, and total coppers are the average concentrations in the first 100 m (in 10^−6^ molCu/m^3^). Total copper represents copper in the dissolved and particulate phases. Legends with a star (*) indicate that the value has been multiplied by 10 in the plot for visualization.

The average Cu:C ratio decreases progressively from 15 to 10 to 8 μmolCu:molC for phytoplankton uptake, zooplankton recycling, and particle remineralization, respectively, and the ensuing modeled export ratio of 16 μmolCu:molC agrees with Semeniuk et al.'s (2016) estimations between 1.5 and 15 for the North Pacific region. We calculated the Cu:C ratio in the dissolved phase from the total organic and inorganic dissolved Cu and P and used the Redfield ratio of 106:1 molC:molP. We found 10 μmolCu:molC in the dissolved phase on average over the surface ocean. Regional Cu budgets and ratios are available in Appendix A (see Figure A1).

## 4 Toward a General View of Copper Biogeochemical Cycling

**Figure 7:**
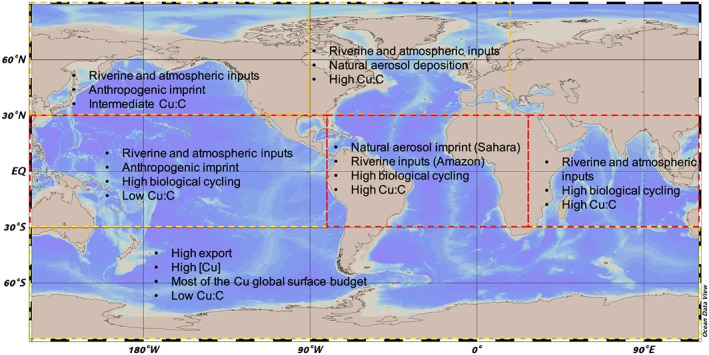
Map of the main processes affecting copper cycling and budgets in the surface ocean.

We can also use our model to highlight the most important processes driving Cu cycling in each ocean region (Figure 7). The impacts of the different anthropogenic and natural Cu external sources on the surface ocean are not evenly distributed, with the northern hemisphere oceans receiving more external inputs of Cu from rivers and aerosols than the southern hemisphere. In particular, the North and Equatorial Atlantic regions receive over 2.5 GmolCu/year from rivers and 0.003 GmolCu/year from aerosol deposition, mostly from the Amazon river and Saharan dust deposition (see Figure A1). The surface Pacific, on the other hand, receives less copper from external sources but is characterized by the dominance of anthropogenic aerosols, mainly industrialized cities around the Pacific coasts of Asia (see, e.g., Uematsu et al., 1983; Wang et al., 2016). Finally, the southern hemisphere oceans are more isolated from external sources and accordingly receive very low Cu external fluxes.

The equatorial regions host most of the surface ocean biological activity and most of the Cu uptake, recycling and remineralization take place here. As most of the surface DCu in the equatorial Pacific is consumed by phytoplankton, the dissolved Cu:C ratio in the water column remains relatively low (7.4 μmolCu:molC; see Figure A1). Surface Cu:C ratios in the Atlantic and Indian oceans are higher (between 15 and 20 μmolCu:molC) than in the Pacific and Southern Ocean (between 7 and 11 μmolCu:molC) due to the higher Cu fluxes from rivers and aerosols.

Despite the reduced external Cu supply rates, about 11 GmolCu are found in the top 100 m of the southern hemisphere oceans, representing approximately 50% of the global surface Cu budget (see Figure A1). This high [DCu] is driven by the intense seasonally variable vertical transport of nutrient‐rich water to the surface (Toggweiler & Samuels, 1995). The Southern Ocean is the main particulate organic carbon export region in the global ocean (Schlitzer, 2002), and the Cu export rate per square meter is 5 to 12 times higher in this region than elsewhere. Once exported below the euphotic layer, Cu slowly sinks to the bottom, and a fraction will be buried into sediments.

Thanks to recent developments, PISCES is the first global biogeochemical model to represent a range of trace metals (Co, Zn, Fe, and Mn; see, e.g., Hulten et al., 2017; Tagliabue et al., 2018) and now Cu. The next developments should include relations between these different elements in phytoplankton cells in order to observe the response of phytoplankton communities to the different elemental ratios in external nutrient sources (Hirose, 2007; Wang et al., 2017). These modeling developments should also be paired with sampling efforts in order to obtain reliable estimates of the trace metal concentrations and elemental ratios in aerosols, rivers, and planktonic cells in various regions of the global ocean.

## 5 Copper Cycling in the OMZs

**Figure 8:**
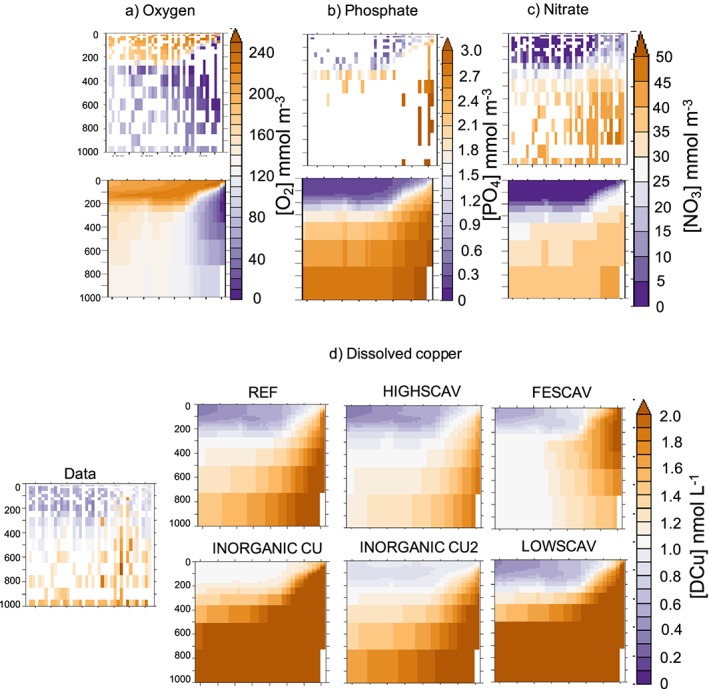
Measured (top) and modeled (bottom) oxygen (a), phosphate (b), and nitrate (c) in the Pacific oxygen minimum zone (GP16 section) in mmol/m^3^. (d) presents [DCu] in the data and in each of the simulations (in nmolCu/L). The cruise transect is shown on Figure 2.

Figures 1 and 2 show that [DCu] in the Pacific OMZ seems overestimated in our model. To evaluate the representation of the Pacific OMZ in PISCES, we use the GEOTRACES GP16 section (Moffett & German, 2018) and compare our model results with the oxygen and nutrients concentrations measured in situ (Figure 8). The modeled oxygen concentration is too high below 200 m, and the expansion of the Pacific OMZ is not well represented: The very low oxygen concentrations (below 50 mmol/m^3^) are constrained between 100 and 300 m in the model, whereas they are observed until 800 m in the measurements (Figure 8a). This feature was already observed by Aumont et al. (2015) who hypothesized that it may be linked with too intense ventilation of oxygen rich waters from the Southern Ocean. However, the macronutrients nitrate and phosphate from PISCES match the GP16 data well, suggesting that their distribution is not being affected by incorrect rates of remineralization (Figures 8b and 8c).

Modeled [DCu] in the Pacific OMZ is at least 0.5 nmolCu/L higher than the measurements across all our simulations (Figure 8d). Only the increased scavenging in HIGHSCAV leads to a decrease of [DCu] in the OMZ and brings the simulated concentrations closer to the measurements. However, the deeper [DCu] becomes underestimated in this simulation (see Figure 2).

Ultimately, our model either contains a too strong Cu source or is missing a Cu sink specific to low oxygen waters. However, there is no sediment source of Cu in the model that could explain the high [DCu] close to the continental shelf, and there is no major river flow or atmospheric deposition flux in this region. It thus appears likely that there is a Cu specific sink operating in low oxygen waters. One candidate may be sulfides, which are very strong scavenging particles (Dyrssen, 1988), and have been measured in the Arabian Sea OMZ (Theberge et al., 1997). Even though there is no evidence of high sulfide concentrations in the Pacific OMZ, Janssen et al. (2014) demonstrated that nutrient‐sulfide precipitation may occur in particles microenvironment. Bianchi et al. (2018) calculated that, in the Pacific OMZ, it could be responsible for the scavenging of 1.4 and 9.7 Gmol/year of Cd and Zn, respectively.

In addition to abiotic sinks, it is possible that high rates of denitrification in OMZs, catalyzed by the Cu‐containing enzyme nitrous oxide reductase (Codispoti et al., 2001; Granger & Ward, 2003), may be a component of Cu removal. Also, denitrification and ammonia oxidation by archea requires Cu as well as Fe (Glass & Orphan, 2012). Our model includes explicit representation of denitrification but does not account for Cu consumption by denitrifying bacteria, which will require two Cu atoms per nitrous oxide reductase. Also, Posacka et al. (2019) hypothesized that up to 50% of biogenic Cu in the ocean could be consumed by bacteria. Therefore, Cu scavenging by sulfides in particle microenvironments as well as Cu uptake by denitrifying bacteria could be a significant component of Cu cycling in OMZs. These processes could be incorporated in future developments of the PISCES model in order to assess their importance.

## 6 Conclusions

This study presents a global 3‐D coupled physical‐biogeochemical model of oceanic Cu cycling, developed within the widely used NEMO/PISCES model. The model captures correctly the main features of Cu distribution in the ocean: low surface concentrations and linear increase with depth. This study brings confirmation that reversible scavenging is the main driver of [DCu] vertical distribution and the scavenging rate determines the slope of the linear profile of [DCu] over the global ocean. Moreover, our simulations support the hypothesis that ligand‐bound copper has to be at least partly bioavailable for phytoplankton to maintain their cellular Cu ratio.

We present a global budget of surface Cu including biogeochemical processes such as uptake, recycling, remineralization, and export, finding that external Cu sources deliver about 7 GmolCu/year to the surface oceans and that phytoplankton uptake represents 31 GmolCu/year. These new estimates provide a shorter residence time than previously calculated (approximately 10 times shorter). Moreover, this new value may be underestimated as some external Cu sources are likely missing in our budget. Equatorial regions are responsible for the majority of the global Cu uptake and biological cycling, whereas the midlatitudes and high latitudes (in particular the southern ocean) are responsible for most of the Cu export below 100 m. The southern part of the ocean also gathers 50% of the global surface Cu budget. Finally, the high Cu:C ratios in the surface Atlantic and Indian oceans seem to be linked with the important Cu fluxes from natural and anthropogenic external sources.

Although our model overestimates [DCu] in the OMZ, this points to the potential role for additional processes associated with particle microenvironments or bacterial cycling in driving the Cu distribution in low oxygen systems. Further developments in the NEMO/PISCES model should also include explicit effects of Cu on phytoplankton growth (fertilizing and toxic effects), interaction effects with other trace metals such as Fe, and a better representation of ligands cycling. These developments should be paired with measurements and experiments to better constrain the model hypotheses.

## Figures and Tables

**Figure C1:**
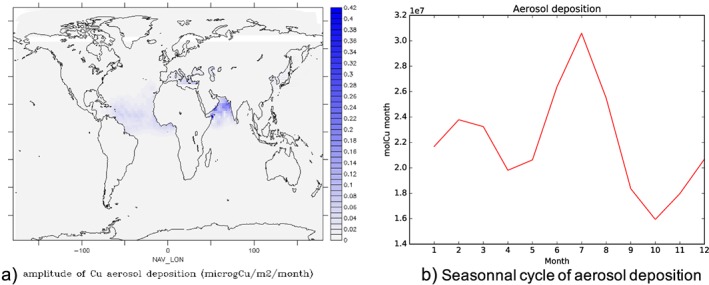
a) Amplitude of average monthly Cu aerosol deposition (*μ*gCu month^−1^). b) Seasonal cycle of average Cu deposition (molCu month^−1^) over the global ocean.

## Appendix A Regional Copper Budgets

### A.1

The northern Pacific and Atlantic regions receive more Cu from rivers than their southern counterparts but receive a similar amount of natural Cu aerosols (Figure A1). Also, the equatorial regions receive most of the riverine Cu (about 2 Gmol/year for both the equatorial Pacific and the equatorial Atlantic). The northern Pacific region receives more Cu from anthropogenic aerosols, mainly because of the north Asian sources. The equatorial Atlantic also receives most of the global natural aerosol load from the Sahara (1 Gmol/year). Also, Cu export is 2 to 5 times more important in the southern regions than in the northern regions, which is mainly linked to physical processes. Moreover, the export values of SCu per unit area are an order of magnitude higher in the southern regions than in the northern ones, making the southern region a more important Cu sink than the equatorial and northern regions. Most of the biological activity is found in the equatorial regions; therefore, most of the Cu uptake, remineralization, and recycling are occurring in the equatorial Pacific and Atlantic. Overall, the southern oceanic regions hold most of the global Cu content. There is in total 8 GmolCu in the top 100 m in the southern Atlantic and Pacific, whereas there is 7 GmolCu in the top 100 m of the equatorial Pacific and Atlantic. However, the average Cu concentrations are higher in the equatorial regions.

The Cu:C of particulate and dissolved Cu is higher in the Atlantic. This is probably linked to the higher Cu fluxes from external sources. Likewise, the Cu:C ratio in particulate export is higher in the Atlantic (19 μmolCu:molC). Also, there is a strong gradient in the Cu:C ratios from the equatorial regions toward the midlatitudes and high latitudes. In particular, for dissolved elements, the Cu:C ratio in the northern regions is between 98 and 117 μmolCu:molC, and it is around 20 to 30 μmolCu:molC in the equatorial regions and around 150 to 250 μmolCu:molC in the southern regions. Likewise, the Cu:C ratio for phytoplankton uptake follows the same increasing trend from equatorial regions to the higher latitudes, showing that phytoplankton adapt to the high Cu ratio in their environment by increasing the Cu:C of their nutrient uptake. However, the Cu:C ratio in remineralization processes decreases toward the poles from, respectively, 8.3 and 11 in the equatorial Pacific and Atlantic and decreases to about 7.3 and 7.7 in the northern and southern Pacific and Atlantic, respectively.

## Appendix B Seasonal Cycling of DCu

### B.1

The Southern Ocean and the Mediterranean are the regions that experience the most intense seasonal variations in surface [DCu] (Figure B1a). In the Southern Ocean, this is probably linked with the very intense changes in surface physical and biogeochemical conditions. In the Mediterranean, the important impacts of external nutrient sources on the surface biogeochemistry may also concur to the important variability we observe. Regions of high productivity and upwelling regions such as the western coasts of Africa and America also have a marked seasonal amplitude of [DCu] between 0.3 and 0.5 nmolCu/L.

Figure B1b shows the global seasonal cycle of surface [DCu]. This figure shows that the seasonal cycling has a weak amplitude on the global scale. However, global [DCu] seems higher during Austral spring (between September and November).

## Appendix C Seasonal Variability of Atmospheric Deposition

### C.1

The most important variability in aerosol Cu deposition is located in the Mediterranean and North Atlantic, downwind of the Sahara, and in the North Indian Ocean downwind of the Sahara and Middle East deserts (Figure C1a).

Figure C1b shows that Cu deposition from aerosols is highly variable over a year. The difference between the lowest deposition flux in October and the highest deposition flux in July is over 0.015 GmolCu/month). Aerosol deposition is a highly dynamic process that varies greatly in space and time. Our model accounts for such variability thanks to the state‐of‐the‐art Cu deposition model from Paytan et al. (2009).
